# Usability evaluation methods employed to assess information visualisations of electronically stored patient data for clinical use: a protocol for a systematic review

**DOI:** 10.1186/s13643-017-0544-1

**Published:** 2017-07-28

**Authors:** Antonia Burt, Lauren Morgan, Tatjana Petrinic, Duncan Young, Peter Watkinson

**Affiliations:** 1 Nuffield Department of Clinical Neurosciences, University of Oxford, Headley Way, Oxford, OX3 9DU UK; 2 Nuffield Department of Surgical Sciences, Level 6, John Radcliffe Hospital, University of Oxford, Headley Way, Oxford, OX3 9DU UK; 3 Bodleian Health Care Libraries, Level 3, Academic Centre, John Radcliffe Hospital, Headington, University of Oxford, Oxford, OX3 9DU UK; 4 Intensive Care and Acute Medicine, Oxford University Hospital NHS Foundation Trust, Headley Way, Oxford, OX3 9DU UK

**Keywords:** User-computer interface, Information visualisation, Usability evaluation, Electronic health record, Clinical decision-making, Human-computer interaction, HCI

## Abstract

**Background:**

The use of electronic records in healthcare is increasing. To avoid errors, it is essential that the data displays used by these systems are usable: efficient, effective and satisfying. A wide variety of display techniques are used to present clinical data, but the best methods to assess the usability of these techniques have not been determined.

This systematic review will answer the question: What methods are employed to assess the usability of electronic visualisations of patient data for clinical use? The results of this systematic review will then be used to inform best assessment and design practice.

**Methods:**

MEDLINE, EMBASE, CINAHL, OpenGrey, and the Cochrane Database of Systematic Reviews will be searched for original studies related to the usability of electronic information visualisations of patient data for clinical use. Reference lists of eligible studies and relevant reviews will be explored to identify further eligible studies.

**Discussion:**

This systematic review will identify methods used to assess the usability of electronic information visualisations of patient data for clinical use. We will summarise the similarities and differences between the methods found. Our results will inform best practice when developing new user interfaces to display electronic patient data for clinical use.

**Trial registration:**

PROSPERO CRD42016041604

**Electronic supplementary material:**

The online version of this article (doi:10.1186/s13643-017-0544-1) contains supplementary material, which is available to authorized users.

## Background

There is an increasing use of electronic patient record (EPR) systems in healthcare. The UK government has a target for a comprehensive electronic health system in England by 2020 [1].

Clinicians use EPR systems to make treatment decisions. It is essential that the information presented in these electronic visualisations is usable—efficient, effective and satisfying. If clinical data are not displayed in a usable format, this can compromise care, for example, by leading to delays in diagnosis or treatment.

A wide variety of visual techniques are used to display clinical data. Usability tools are used for their assessment. How and why individual tools have been used has not been studied. Given the heterogeneity of systems, it is unlikely that one assessment tool would allow accurate assessment or comparison of them all.

A systematic review published in 2014 looked at the use of visualisation techniques and evaluated innovative approaches to information visualisation of electronic health record data [2]. Whilst this review acknowledged the need for a usable design (one of the themes identified), it did not look at the tools and techniques to assess the usability of the different information visualisation techniques.

### Aim

This systematic review aims to identify and systematically review the methods used to assess the usability of information visualisations of patient data for clinical use. The review will for the first time summarise the methods used to assess usability of information visualisations of individual patient data for clinical use. The strengths and weaknesses found for different techniques in different situations will be discussed. This will allow those developing such software to undertake development in light of the assessment methods that they should use to ensure quality software. It will also allow customers to ensure products they require are suitably assessed and meet their needs.

## Methods/design

This protocol adheres to the requirements of Preferred Reporting Items for Systematic Review and Meta-analysis Protocols (PRISMA-P) [3] which is included as Additional file 1.

### Eligibility criteria

Studies satisfying all the criteria below will be included:

- Describes a method of electronic information visualisation used to display patient data AND includes an assessment of the information visualisation
- Is related to electronic visualisations either prototype or used in clinical practice
- Is related to methods for evaluating usability of the visualisation technique used to display the medical data
- Displays individual patient data, not just patient cohort data
- Is for clinician’s use only and not for patient access
- Is not related to dentistry

### Exclusions

Systematic reviews will not be included in the review, but appropriate studies referenced in reviews will be included. Correspondence and short communications will be excluded. Doctoral research will be included. As our systematic review aims to identify which methods have been used and where these were informative, we will not include protocols without usage data.

### Study design

The study design is a systematic search of the medical literature followed by a narrative synthesis of the results.

### Setting

Individual patient data display is implemented in any healthcare settings where it is used for clinical use.

### Time frame

There will be no restriction placed on the time frame of the studies.

### Years considered

Studies published from 1996 onwards will be considered.

### Language

No language restrictions will be applied.

### Information sources

Literature search strategies will be developed using Medical Subject Headings (MeSH) and text words related to the usability of information visualisation methods for displaying patient medical data for clinical use.

The following databases will be searched: MEDLINE (Ovid), EMBASE (Ovid), CINAHL (HDAS), OpenGrey and the Cochrane Database of Systematic Reviews (Wiley).

Reference lists of eligible studies and relevant reviews will be explored to identify further eligible studies.

### Search strategy

A draft of the search strategy was developed by three of the authors (AB, LM and TP, a medical librarian). The proposed search strategy is shown in Additional file 2.

## Study records

### Data management

The citations and full text of papers identified from the search will be stored using Mendeley. A data extraction form will be developed in Excel and piloted.

Literature search results will be uploaded to Covidence, an online software program designed to improve the production of systematic reviews.

### Selection process

Two assessors will independently screen title and abstracts of papers returned by the search against the inclusion criteria. If there is uncertainty from the abstract, the full text will be reviewed. Papers selected on title and abstract will be full-text screened for eligibility. Eligible papers will be included in the study.

Disagreements about eligibility will be resolved by a third party. We will record the reasons for excluding the studies.

The study selection process is illustrated by the Preferred Reporting Items for Systematic Reviews and Meta-analyses (PRISMA) flow diagram in Additional file 3.

### Data collection process

Data extraction forms will be created using Excel and piloted prior to use. Two reviewers will independently extract data from the full text of eligible papers. Any uncertainties regarding data extraction will be resolved by discussion amongst the authors.

### Data items extracted

We will extract the following data items from each publication:

Study characteristics:

- Date of study

Period of data collection

- Type of study design

Patient demographics

- Clinician/users of system demographics

Study setting

- Type of task visualised

Outcome measures

- Country of study

Display data:

Information contained in the display, including for example:

- Static: patient demographics, admission history, past medical history
- Semi static: ward/bed number
- Dynamic: laboratory data, vital signs, neurological status, respiratory status, cardiovascular status, assessment/warning scores, drug charts, fluid charts and medical notes of various clinician groups

Visualisation techniques/display categories as described by Starren et al. [4]:

- List (simple/nested)

Table

- Graph (simple chart/configural chart/graph notation)

Icons (atomic icon/iconic language)

- Generated text

The following display design features will be identified and recorded:

- Interaction level: display, input, alerting and messaging capabilities

Some publications may refer to more than one category of visualisation technique or type of information. Multiple data points will be captured where relevant. The pilot phase of the data extraction form may identify other relevant information which will also be collected.

### Usability data

We will extract data on how usability was assessed in the following categories:

- Usability assessment technique/tool type

Usability assessment

- Test/retest performance of tool

Inter-rater performance of tool

- Outcome scale generated

Any assessment of the tool’s user-friendliness

- Specific data required by the tool

We will also extract data, where available, on whether the tool records the following categories suggested by Kopanitsa et al. [5]:

- Efficiency: time to complete tasks (% of tasks fully completed, % of tasks half completed); comparing task completion quality using software/new software compared to without software/previous software
- Effectiveness: % of errors; surveys on % of participants’ responses to task completion; comparing task completion quality using software/new software compared to without software/previous software
- Satisfaction: % of participants who make positive/negative comments about the system

This list of performance metrics is not exhaustive, and other measures identified in the literature will also be used.

### Outcomes and prioritisation

The primary outcome will be to document methods employed to assess the usability of electronic information visualisations of patient data for clinical use.

Secondary outcomes will include a narrative comparison of the identified methods.

### Risk of bias of individual studies

The methodological quality of the studies will be assessed using a modified Downs and Black (D&B) checklist [6]. This checklist was designed to provide an evaluation of the quality of both randomised and non-randomised studies of healthcare interventions on the same scale.

The D&B checklist will be modified to create a 20-question checklist by omitting questions 5, 9, 12, 14, 17, 25 and 26. These questions are deemed not appropriate for assessing the methodological quality of the studies which will be evaluated.

### Data synthesis

We anticipate that the data extracted will be most appropriate for qualitative synthesis. This will be presented as a narrative synthesis. Themes will be identified in the text, and tables will be used to summarise and explain the characteristics/findings of the included studies.

## Discussion

Currently, there are a variety of information visualisation methods used to present electronic medical data for clinical use [2]. However, there is minimal evidence in the literature regarding assessments of the usability of these systems, despite a clear need to be able to do this effectively.

To date, we are unaware of any systematic review exploring the methods used to assess the usability of electronic information visualisations of patient data for clinical use. This review will summarise the similarities and differences between methods used to assess the usability of electronic information visualisations of patient data for clinical use. We will present the differences between the methods found. Our results will inform best practice when developing new user interfaces to display electronic patient data for clinical use.

### Limitations

The systemic review will be limited by the quality of the data available.

## Additional files

Additional file 1: PRISMA-P checklist.doc. This file contains the completed PRISMA-P checklist. (DOC 102 kb)

Additional file 2: Search strategy table.doc. This file contains an example MEDLINE search strategy. (DOCX 21 kb)

Additional file 3: PRISMA 2009 flow diagram.doc. This file contains the PRISMA 2009 flow diagram. (DOC 47 kb)

## Abbreviations

CINAHL: Cumulative Index to Nursing and Allied Health Literature
EMBASE: Excerpta Medica database
EPR: Electronic patient record
MEDLINE: Medical Literature Analysis and Retrieval System Online
MeSH: Medical Subject Headings
