# Supplementary material for: Usability evaluation methods employed to assess information visualisations of electronically stored patient data for clinical use: a protocol for a systematic review
Source: Syst Rev. 2017 Jul 28;6:148. doi: 10.1186/s13643-017-0544-1 (PMC5534029; doi:10.1186/s13643-017-0544-1)
Supplement: Supplementary file 1 — PRISMA-P checklist.doc. This file contains the completed PRISMA-P checklist. (DOC 102 kb) [file 13643_2017_544_MOESM1_ESM.doc]

**Additional file 1**

**PRISMA-P (Preferred Reporting Items for Systematic review and Meta-Analysis Protocols) 2015 checklist: recommended items to address in a systematic review protocol***

| Section and topic | Item No | Checklist item |
| --- | --- | --- |
| ADMINISTRATIVE INFORMATION | | |
| Title: |  |  |
| Identification | 1a | Identify the report as a protocol of a systematic review  **Usability Evaluation Methods used to assess Information Visualisations of Patient Data for Clinical Decision Making: A protocol for a systematic review.** |
| Update | 1b | If the protocol is for an update of a previous systematic review, identify as such  **The protocol is not an update of a previous systematic review** |
| Registration | 2 | If registered, provide the name of the registry (such as PROSPERO) and registration number  **PROSPERO – reference: CRD42016041604 Registered on 4^th^ July 2016** |
| Authors: |  |  |
| Contact | 3a | Provide name, institutional affiliation, e-mail address of all protocol authors; provide physical mailing address of corresponding author  **Antonia Burt**  Kadoorie Centre: Critical Care Research and Education Centre  Level 3, John Radcliffe Hospital  Headley Way  Oxford  OX3 9DU  Email: [antonia.burt@ndcn.ox.ac.uk](mailto:antonia.burt@ndcn.ox.ac.uk)  **Lauren Morgan**  Kadoorie Centre: Critical Care Research and Education Centre  Level 3, John Radcliffe Hospital  Headley Way  Oxford  OX3 9DU  Email: [lauren.morgan@nds.ox.ac.uk](mailto:lauren.morgan@nds.ox.ac.uk)  **Tatjana Petrinic**  University of Oxford  Bodleian Health Care Libraries  Level 3, Academic Centre  John Radcliffe Hospital  Headington  Oxford  OX3 9DU  Email: [tatjana.petrinic@bodleian.ox.ac.uk](mailto:tatjana.petrinic@bodleian.ox.ac.uk)  **Duncan Young**  Kadoorie Centre: Critical Care Research and Education Centre  Level 3, John Radcliffe Hospital  Headley Way  Oxford  OX3 9DU  Email: [duncan.young@ndcn.ox.ac.uk](mailto:duncan.young@ndcn.ox.ac.uk)  **Peter Watkinson (corresponding author)**  Kadoorie Centre: Critical Care Research and Education Centre  Level 3, John Radcliffe Hospital  Headley Way  Oxford  OX3 9DU  Email: [peter.watkinson@ndcn.ox.ac.uk](mailto:peter.watkinson@ndcn.ox.ac.uk) |
| Contributions | 3b | Describe contributions of protocol authors and identify the guarantor of the review  **Antonia Burt** (first author)  **Lauren Morgan** (developing data extraction criteria)  **Tatjana Petrinic** (developing initial search strategy)  **Duncan Young** (advised on the development of the protocol and revised the manuscript)  **Peter Watkinson** (guarantor of the review and corresponding author)  All authors read, provided feedback and approved the final manuscript. |
| Amendments | 4 | If the protocol represents an amendment of a previously completed or published protocol, identify as such and list changes; otherwise, state plan for documenting important protocol amendments  **This protocol is not an amendment. If an amendment of the protocol is required, the date of each amendment along with a description of the change and rationale for the amendment will be provided.** |
| Support: |  |  |
| Sources | 5a | Indicate sources of financial or other support for the review  **This systematic review protocol is funded by the Department of Health and Wellcome Trust through the Health Innovation Challenge Fund. This publication presents independent research commissioned by the Health Innovation Challenge Fund (HICF-R9-524; WT-103703/Z/14/Z), a parallel funding partnership between the Department of Health and Wellcome Trust.** |
| Sponsor | 5b | Provide name for the review funder and/or sponsor  **The University of Oxford is the sponsor** |
| Role of sponsor or funder | 5c | Describe roles of funder(s), sponsor(s), and/or institution(s), if any, in developing the protocol  **The University of Oxford assumes the ultimate responsibility for the initiation, management and financial control for the research project.** |
| INTRODUCTION | | |
| Rationale | 6 | Describe the rationale for the review in the context of what is already known  **There is little guidance from the research literature on what are the best methods to assess the usability of information visualisation techniques. Please see Background section for further detail.** |
| Objectives | 7 | Provide an explicit statement of the question(s) the review will address with reference to participants, interventions, comparators, and outcomes (PICO)  **What methods are used to assess the usability of information visualisations of patient data for clinical decision making? Please see Methods/ Design section – subsection Aims** |
| METHODS | | |
| Eligibility criteria | 8 | Specify the study characteristics (such as PICO, study design, setting, time frame) and report characteristics (such as years considered, language, publication status) to be used as criteria for eligibility for the review  **Please see Methods/ Design section – subsection Eligibility criteria** |
| Information sources | 9 | Describe all intended information sources (such as electronic databases, contact with study authors, trial registers or other grey literature sources) with planned dates of coverage  **Please see Methods/ Design section – subsection Information sources** |
| Search strategy | 10 | Present draft of search strategy to be used for at least one electronic database, including planned limits, such that it could be repeated  **Please see Methods/ Design section – subsection Search strategy** |
| Study records: |  |  |
| Data management | 11a | Describe the mechanism(s) that will be used to manage records and data throughout the review  **Please see Methods/ Design section – subsection Study records** |
| Selection process | 11b | State the process that will be used for selecting studies (such as two independent reviewers) through each phase of the review (that is, screening, eligibility and inclusion in meta-analysis)  **Please see Methods/ Design section – subsection Selection Process** |
| Data collection process | 11c | Describe planned method of extracting data from reports (such as piloting forms, done independently, in duplicate), any processes for obtaining and confirming data from investigators  **Please see Methods/ Design section – subsection Data collection process** |
| Data items | 12 | List and define all variables for which data will be sought (such as PICO items, funding sources), any pre-planned data assumptions and simplifications  **Please see Methods/ Design section – subsection Data items** |
| Outcomes and prioritization | 13 | List and define all outcomes for which data will be sought, including prioritization of main and additional outcomes, with rationale  **Criteria for establishing how to assess the usability of an information visualisation on an electronic display to support clinical decision making will be the primary outcome.** |
| Risk of bias in individual studies | 14 | Describe anticipated methods for assessing risk of bias of individual studies, including whether this will be done at the outcome or study level, or both; state how this information will be used in data synthesis  **The methodological quality of the studies will be assessed using a modified Downs and Black (D&B) checklist. Please see Methods/ Design section – subsection Risk of bias of individual studies** |
| Data synthesis | 15a | Describe criteria under which study data will be quantitatively synthesised  **It is anticipated the data extracted will be most appropriate for qualitative synthesis.** |
|  | 15b | If data are appropriate for quantitative synthesis, describe planned summary measures, methods of handling data and methods of combining data from studies, including any planned exploration of consistency (such as I^2^, Kendall’s τ)  **Not applicable as data not appropriate for quantitative synthesis** |
|  | 15c | Describe any proposed additional analyses (such as sensitivity or subgroup analyses, meta-regression)  **Not applicable** |
|  | 15d | If quantitative synthesis is not appropriate, describe the type of summary planned  **This will be presented descriptively in the text and tables to summarise and explain the characteristics/ findings of the included studies.** |
| Meta-bias(es) | 16 | Specify any planned assessment of meta-bias(es) (such as publication bias across studies, selective reporting within studies)    **This will not be included** |
|  |  |  |
| Confidence in cumulative evidence | 17 | Describe how the strength of the body of evidence will be assessed (such as GRADE)  **The modified Downs & Black checklist will be used to assess the strength of the body of evidence** |

*** It is strongly recommended that this checklist be read in conjunction with the PRISMA-P Explanation and Elaboration (cite when available) for important clarification on the items. Amendments to a review protocol should be tracked and dated. The copyright for PRISMA-P (including checklist) is held by the PRISMA-P Group and is distributed under a Creative Commons Attribution Licence 4.0.**

*From: Shamseer L, Moher D, Clarke M, Ghersi D, Liberati A, Petticrew M, Shekelle P, Stewart L, PRISMA-P Group. Preferred reporting items for systematic review and meta-analysis protocols (PRISMA-P) 2015: elaboration and explanation. BMJ. 2015 Jan 2;349(jan02 1):g7647.*
