# Supplementary material for: Usability evaluation methods employed to assess information visualisations of electronically stored patient data for clinical use: a protocol for a systematic review
Source: Syst Rev. 2017 Jul 28;6:148. doi: 10.1186/s13643-017-0544-1 (PMC5534029; doi:10.1186/s13643-017-0544-1)
Supplement: Supplementary file 2 — Search strategy table.doc. This file contains an example MEDLINE search strategy. (DOCX 21 kb) [file 13643_2017_544_MOESM2_ESM.docx]

**Additional file 1 – Example search strategy for MEDLINE database**

| Search strategy for MEDLINE database |
| --- |
| 1. USER-COMPUTER INTERFACE/ |
| 1. “user interface*”.ti,ab |
| 1. “graphical user interface*”.ti,ab |
| 1. “dashboard*”.ti,ab |
| 1. 1 OR 2 OR 3 OR 4 |
| 1. COMPUTER GRAPHICS/ |
| 1. “computer graphic*”.ti,ab |
| 1. “information visualization*”.ti,ab |
| 1. “data visualization*”.ti,ab |
| 1. “visual analytic*”.ti,ab |
| 1. 6 OR 7 OR 8 OR 9 OR10 |
| 1. MEDICAL RECORD SYSTEMS, COMPUTERIZED/ |
| 1. “medical record*”.ti,ab |
| 1. “patient record*”.ti,ab |
| 1. “Integrated information”.ti,ab |
| 1. ELECTRONIC HEALTH RECORDS/ |
| 1. “electronic health record*”.ti,ab |
| 1. “electronic medical record*”.ti,ab |
| 1. “charting system*”.ti,ab |
| 1. 12 OR 13 OR 14 OR 15 OR 16 OR 17 OR 18 OR 19 |
| 1. MEDICAL INFORMATICS/ |
| 1. “big data”.ti,ab |
| 1. “healthcare data”.ti,ab |
| 1. “health care data”.ti,ab |
| 1. (“medical informatics” AND application*).ti,ab |
| 1. 21 OR 22 OR 23 OR 24 OR 25 |
| 1. usability.ti,ab |
| 1. “usability evaluation”.ti,ab |
| 1. Evaluation.ti,ab |
| 1. 27 OR 28 OR 29 |
| 1. 5 OR 11 |
| 1. 20 OR 26 |
| 1. 31 AND 32 |
| 1. 33 AND 30 |
| 1. 34 [limited to:Publication Year 1996-2016] |
