# Supplementary material for: Usability evaluation methods employed to assess information visualisations of electronically stored patient data for clinical use: a protocol for a systematic review
Source: Syst Rev. 2017 Jul 28;6:148. doi: 10.1186/s13643-017-0544-1 (PMC5534029; doi:10.1186/s13643-017-0544-1)
Supplement: Supplementary file 3 — PRISMA 2009 flow diagram.doc. This file contains the PRISMA 2009 flow diagram. (DOC 47 kb) [file 13643_2017_544_MOESM3_ESM.doc]

**Additional file 2 – Preferred Reporting Items for Systematic Reviews and Meta-Analyses (PRISMA) Flow Diagram**

Records identified through database searching
(n = )

Additional records identified through other sources
(n = )

Total number of records identified before duplicates removed (n= )

Duplicates removed

(n= )

Records to be title and abstract screened

(n= )

Records excluded

(n= )

Full-text articles assessed for eligibility (n= )

Full-text articles excluded, with reasons (n = )

Not about data visualisation (n= )

Display has no data visualisation (n= )

Data visualisation not usability evaluated (n= )

Not patient medical data (n= )

Not for clinical decision making (n= )

Not displaying individual patient data (n= )

Not for clinician use only (n= )

Written before 1996 (n= )

Not original research (n= )

Not a journal article (n= )

Eligible studies included in data extraction phase

(n= )
